# Sociodemographic and Mental Health Characteristics of Adults with Different Gender Trajectories

**DOI:** 10.1007/s10508-026-03475-5

**Published:** 2026-06-03

**Authors:** Pablo Expósito-Campos, Karmele Salaberria, José Ignacio Pérez-Fernández

**Affiliations:** 1 Department of Clinical and Health Psychology and Research Methods, Faculty of Psychology, University of the Basque Country, Tolosa Hiribidea 70, 20018 Donostia-San Sebastián, Gipuzkoa Spain; 2 Predoctoral Research Fellowship Program of the Department of Education of the Government of the Basque Country, Vitoria-Gasteiz, Spain

**Keywords:** Transgender, Detransition, Gender trajectory, Mental health, Psychological support

## Abstract

There is growing interest in the experiences of transgender and gender diverse (TGD) people and those who discontinue or reverse their gender transition, known as detransition. While an understanding of their similarities and differences could inform better psychological support, no study has simultaneously recruited and compared both groups. In this study, we describe and compare two groups of 29 TGD (*M*_*age*_ = 28.28, 72.4% assigned female at birth) and 22 detransitioning participants (*M*_*age*_ = 28.73, 63.6% assigned female at birth). The criterion for categorizing the detransition group was stopping, shifting, or reversing gender transition alongside a change in gender self-conceptualization. Participants completed two short online surveys and took part in two face-to-face assessments covering their transition/detransition history, including the steps taken during the process, and self-reported mental health, including diagnoses, psychopharmacological medication, and adverse life experiences. All reported social transition, 74.5% administrative transition, and 84.3% medical transition. Detransitioning participants started medical transition at a significantly younger age. In addition, there was a higher proportion of detransitioning participants who reported side/unwanted effects of gender-affirming hormonal treatment, and of TGD participants with interest in further medical interventions. No significant differences were found for other variables. A high percentage of participants in both groups self-disclosed a history of poor mental health and adverse life experiences. The findings highlight the diversity of trajectories following gender transition, the difficulty of predicting them, and their importance for informing future mental health services and support.

## Introduction

Transgender and gender diverse (TGD) people experience a mismatch between their sex assigned at birth and their gender identity, referred to as gender incongruence in the 11th edition of the World Health Organization (WHO) *International Classification of Diseases* (WHO, 2022). In many cases, this incongruence leads to significant distress, clinically referred to as gender dysphoria (GD) (American Psychiatric Association, 2022), which TGD people may seek to alleviate through a process of gender transition. Gender transition refers to the process of aligning one’s gender expression and/or physical characteristics with one’s gender identity. This process may involve a series of social, administrative, and/or medical interventions commonly referred to as gender-affirming (Coleman et al., 2022). Social transition may include changes in name, pronouns, clothing, or other non-medical practices (e.g., chest binding or tucking); administrative transition may include applying for a change in the name/gender marker appearing on official documents; and medical transition may include gender-affirming hormonal treatment (GAHT) and various types of genital and non-genital gender-affirming surgery (GAS). These steps are highly individualized; TGD people may choose to undergo some or all of these measures, and not necessarily in a linear order. Therefore, none is essential nor is there a required combination of steps to validate a person’s gender transition. The most recent version of the World Professional Association for Transgender Health standards of care (Coleman et al., 2022) emphasizes the importance to the quality of life of TGD people.

Over the last decade, there has been a significant increase in public and clinical interest in the health experiences and needs of TGD people (Delli & Livas, 2021; Sweileh, 2018). In part, this has been driven by the increasing numbers of people either self-identifying as TGD (Fisher et al., 2024; Twenge et al., 2025) or seeking medical transition, as reported by specialized gender identity services in Western countries (Kaltiala et al., 2020; Wiepjes et al., 2018; Zhang et al., 2021) and other healthcare organizations (Sun et al., 2023). The largest increases have been observed among adolescents and young adults, particularly those assigned female at birth (AFAB) relative to those assigned male at birth (AMAB) (Fisher et al., 2024; Sun et al., 2023; Twenge et al., 2025). In addition, some studies have noted an increase in the prominence of TGD people in the media with more positive representations (Bracco et al., 2024), which has been shown to influence people’s perceptions and attitudes (Gillig et al., 2018), and thus their interest in the topic. These trends have also been observed in the Spanish-speaking context, where there has been an increase in referrals for gender-affirming care, mostly from adolescents and young adults AFAB (Expósito-Campos et al., 2023a); a steady increase in biomedical and psychosocial research on TGD populations (Barrientos et al., 2024; Gómez-Gil et al., 2020); and a trend towards more positive and diverse representation in the media (Olveira-Araujo, 2023). This highlights the existence of a social climate that is more aware, respectful, and willing to respond to the specific health needs of the TGD population. However, this increased awareness has coincided with a significant rise in hostile political discourse and restrictive government policies in some parts of the world in recent years (Transgender Europe, 2025; Trans Legislation Tracker, 2025).

Despite the overall progress, TGD people continue to experience a significant burden of mental health problems. In a large systematic review, Pinna et al. (2022) reported consistent findings of increased prevalence of mental health disorders compared to the general population across 165 studies published in the last two decades, primarily from Western, high-income countries, including depression and anxiety, eating disorders, posttraumatic stress disorder (PTSD), personality disorders, substance use disorders, and neurodevelopmental disorders (autism spectrum disorder [ASD] and attention deficit hyperactivity disorder [ADHD]). Other studies have observed a higher prevalence of psychopharmacological medication use, particularly antidepressants and anxiolytics (Kalayjian et al., 2024). In addition, there is evidence of increased prevalence of experiences of bullying (Witcomb et al., 2019), abuse (Strauss et al., 2020), interpersonal (physical or sexual) violence (McLellan et al., 2026), non-suicidal self-injury (Marshall et al., 2016a), suicidal ideation and attempts (Kohnepoushi et al., 2023), and psychiatric hospitalizations (Khanijow et al., 2024). Similar findings have been reported in research with Spanish-speaking TGD populations (Caballero et al., 2025; Castelo-Branco et al., 2021; Ciria-Barreiro et al., 2021; Devís-Devís et al., 2017; García-Vega et al., 2018; Marshall et al., 2016b; Modrego Pardo et al., 2021).

People who undertake gender transition processes show wide variability in their trajectories and decision-making (Koehler et al., 2025). Among these, some choose to discontinue or reverse some or all the social, administrative, and medical aspects associated with the process. This phenomenon is generally referred to as gender detransition, and the discontinuation or reversal of each of the steps taken during gender transition is referred to as social detransition, administrative detransition, and medical detransition, respectively. However, there is considerable heterogeneity in the definition and conceptualization of detransition (Expósito-Campos et al., 2023c; Sanders et al., 2023; Walls et al., 2025), and several, sometimes conflicting, proposals can be found in the literature (Walls et al., 2025). For example, Hildebrand-Chupp (2020) suggested using “detransition” as an umbrella term that includes individuals who take observable actions to return in some way to a pre-transition state (e.g., discontinue GAHT), who change how they understand or conceptualize their identity (e.g., stop identifying as TGD), and who have a negative transition experience (e.g., regret). In contrast, Turban et al. (2022) cautioned against using the broad label of “detransition” to refer to these qualitatively distinct phenomena. Findings from recent studies suggest that these three aspects (i.e., detransition as an act, an identity, and a negative transition experience) often overlap (Littman, 2021; MacKinnon et al., 2022; Pullen Sansfaçon et al., 2023; Vandenbussche, 2022), making it difficult to draw clear boundaries. Other proposals have focused on specific aspects of the experience of detransitioning (cessation/continuation of a TGD identity; Expósito-Campos, 2021; presence or absence of regret; Janssen, 2021) or on the reasons that lead to the discontinuation or reversal of gender transition (Graham, 2017; MacKinnon et al., 2025; Turban et al., 2021). Walls et al. (2025) propose the framework of “gender transition interruptions,” distinguishing specifically between those who reidentify with their sex assigned at birth and those who do not, including those who shift to a different gender identity (e.g., from binary to nonbinary). Our use of the term “detransition” in this article includes participants with diverse gender trajectory outcomes who share the experience of having stopped, shifted, or reversed their gender transition alongside a shift in their gender self-conceptualization, in an attempt to be consistent with the range of experiences reported in previous detransition research (e.g., Littman, 2021; MacKinnon et al., 2022; Pullen Sansfaçon et al., 2023). Regarding prevalence, a recent systematic review found highly heterogeneous rates, with point-prevalence proportions ranging from 1% to 9.8% across studies (Feigerlova, 2025). However, the review also concluded that the overall quality of the available evidence is very low, limited by heterogeneous definitions, retrospective designs, and insufficient follow-up (see also Cohn, 2023).

Although research on detransition is still emerging, findings from community-based surveys and qualitative interview-based studies show some similarities with findings from research conducted with TGD populations. Consistent with reports from gender identity services, the majority appear to be AFAB young adults (Haarer, 2022; Littman, 2021; Littman et al., 2024; MacKinnon et al., 2022; Maragos et al., 2025; O’Donnell, 2023; Pullen Sansfaçon et al., 2023; Vandenbussche, 2022). There is also a high prevalence of co-occurring mental health problems. For example, participants in Littman’s (2021) study showed elevated rates of trauma, depression, anxiety, ADHD, and PTSD. Similarly, Vandenbussche (2022) reported a high prevalence of diagnosed mental health conditions among the study participants, particularly depression, anxiety, PTSD, ADHD, ASD, eating disorders, and personality disorders. In another community-based survey, Littman et al. (2024) found that the average number of lifetime diagnoses received by participants was 3.7, with depression, anxiety, eating disorders, ADHD, ASD, PTSD, and bipolar disorder being among the most common. The authors also reported that 79% of participants had ever engaged in non-suicidal self-injury, and many also reported multiple adverse childhood events and other traumatic experiences. Gould et al. (2024) reported a high prevalence of ASD, ADHD, borderline personality disorder, and general mental health complaints in their sample of detransitioners. Two studies conducted in the Spanish-speaking context tentatively support these findings (Gómes-Porras et al., 2020; Pazos Guerra et al., 2020).

The burden of mental health problems experienced by both TGD people and people who detransition is concerning and deserves much attention. However, while the last decade has seen a progressive development of affirmative psychological interventions for TGD people (Expósito-Campos et al., 2023b), there are no specialized clinical resources for people who detransition, highlighting the need for further research and services (Butler & Hutchinson, 2020; Entwistle, 2021; Irwig, 2022; MacKinnon et al., 2023). In addition, it is unclear whether psychological frameworks and practice approaches for TGD people are appropriate for working with people who detransition. Particularly in the Spanish-speaking context, the limited previous research on the experiences and needs of both populations poses an important challenge for the development of psychological care recommendations, supports, and services.

### The Current Study

For these reasons, we conducted NORTASUN (Basque word for “identity”), a mixed-methods exploratory study of the experiences of gender transition and detransition among Spanish-speaking adults. The overall goal of the project was to better understand participants’ experiences and needs to improve psychological support and care services. It consisted of (1) a qualitative component using semi-structured interviews to understand participants’ lives, experiences, and needs; and (2) a quantitative component using self-report questionnaires to explore their gender-related experiences, mental health, and quality of life. Given the lack of research comparing the experiences of TGD individuals and those who detransition, the primary purpose of this article is to describe and compare these two groups regarding their sociodemographic characteristics, gender-related experiences, and mental health. The research question guiding this study was: To what extent do TGD individuals and individuals who detransition differ across these three variables? These data allow for a better characterization and understanding of the target populations, which is key to any effort to develop mental health care resources. To our knowledge, this is the first study to recruit, analyze, and compare TGD people and people who have detransitioned simultaneously. Therefore, given the lack of prior research to build on and the exploratory nature of the study, we refrained from formulating pre-specified hypotheses about the results of the analysis.

## Method

### Participants and Procedure

**Fig. 1:**
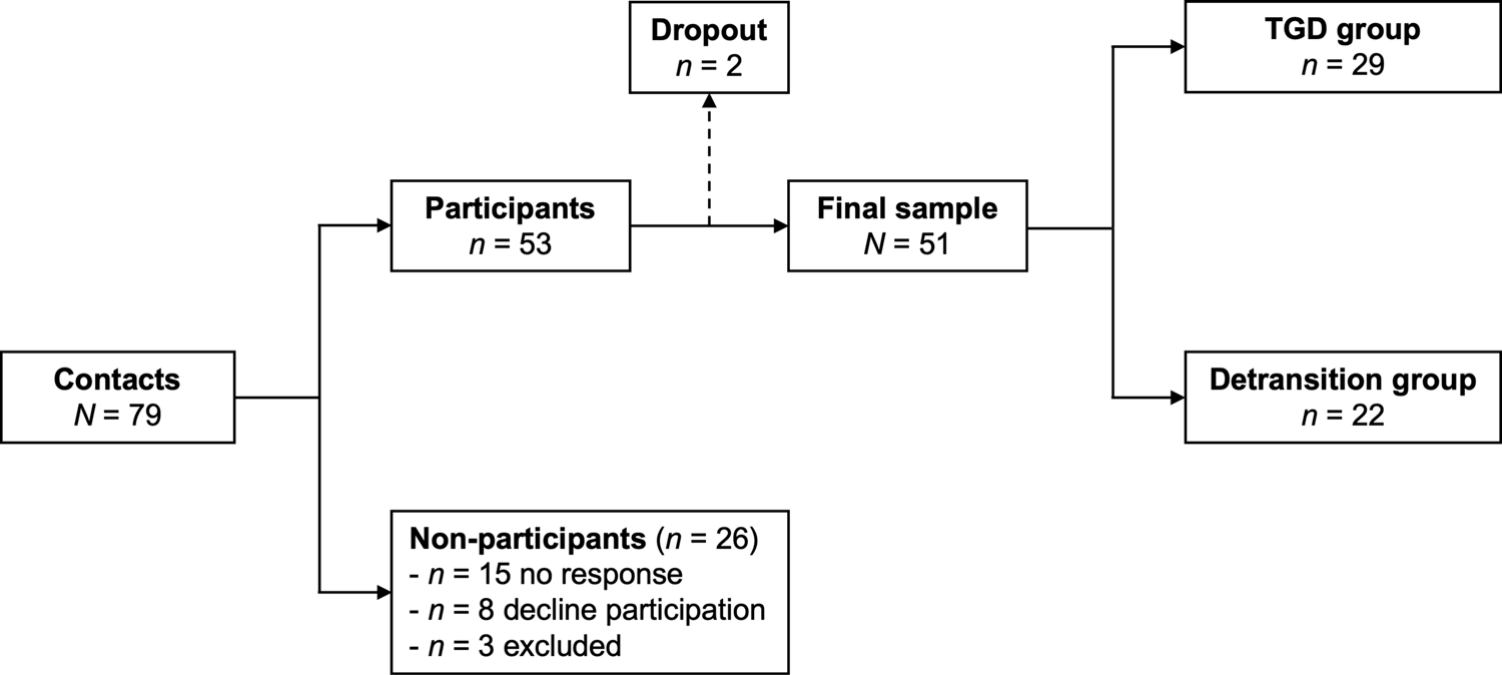
Participant flow chart

Eligibility for the study was based on the following inclusion criteria: (1) currently self-identifying as TGD or as having detransitioned, regardless of the type of gender transition undertaken (social, medical, or both); (2) being 18 years of age or older; and (3) living in a Spanish-speaking country and being fluent in Spanish. Participants were excluded if, at initial contact with the research team, they presented with a mental health condition that could significantly compromise their well-being by participating (e.g., active suicidal ideation), had a physical condition that interfered with their ability to complete the assessment (e.g., chronic throat pain), or if they did not have sufficient availability to participate. Between January 2023 and June 2024, 79 people expressed interest in the study. Of these, three were excluded after the initial contact with the research team. The remaining 76 eligible individuals were formally invited to participate. From this group, 53 people consented to take part (a participation rate of 69.7% among those invited). Two participants, both from the detransitioning group, later withdrew their consent after completion, leaving a final sample of 51 for analysis. The 26 individuals who did not participate included the three who were excluded, 15 who did not respond to the invitation, and eight who declined to participate (see Fig. 1). Those who declined primarily cited reasons related to the emotional burden of revisiting past experiences, not feeling psychologically prepared, or discomfort with the interview format.

As one of the main aims of our study was to make comparisons between TGD people and people with an experience of detransition, we divided our analytical sample into two distinct groups: a “TGD group” and a “detransition group.” The detransition group assignment was based on participant’s accounts of whether they had stopped, shifted, or reversed some or all of the social, administrative, and/or medical aspects of their gender transition, combined with the presence or absence of a self-reported change in their gender self-conceptualization after transitioning. In the absence of comparable studies, this decision was aligned with previous research and the recognition that detransition typically involves a change in identity or self-understanding (Hildebrand-Chupp, 2020; Littman, 2021; MacKinnon et al., 2025; Pullen Sansfaçon et al., 2024; Vandenbussche, 2022). Thus, the first group (TGD) consisted of participants who reported no changes in their gender self-conceptualization after transition (*n* = 29), while the second group (detransition) consisted of participants who stopped, shifted, or reversed transition-related changes and reported a shift in their gender self-conceptualization after transition (*n* = 22).

Given that TGD people, and especially those who have detransitioned, are a hard-to-reach population, we used several strategies identified in the literature to overcome sampling barriers. These included snowball sampling, sampling through community organizations, facility-based sampling, and respondent-driven sampling (Bonevski et al., 2014; Ellard-Gray et al., 2015; Shaghaghi et al., 2011). To promote the study, we created a website that included a detailed explanation of the study’s aims, target population, and methodology, as well as information about the members of the research team, including their professional and educational backgrounds. Through the website, people interested in participating in the study could access a short online registration survey, where they could indicate their interest in participating in the study and their preferred method of contact with the research team. In addition to the website, we created recruitment flyers that were shared on X/Twitter and Instagram through accounts created specifically for the purpose of the study. These flyers included basic information about the study and a QR code that directed interested individuals to the short online registration survey, as well alternative contact options. The study was also promoted on a Reddit subreddit (*r/detrans*) known to be frequented by members of part of the target population. Finally, invitation emails were sent to several regional and national Spanish LGBTQ+ organizations, gender identity units in hospitals that provide gender-affirming care to TGD people, and other providers with experience in transgender health known to the research team.

All individuals who expressed an interest in participating in the study were contacted directly by the research team to provide a detailed explanation of the study. In particular, we informed them about the purpose, design, and organization of the study, the expected time and commitment required for participation, and the potential inconveniences and benefits of participation. Additionally, participants were given a detailed explanation of the anonymous and confidential nature of their participation, data handling and processing, and their rights as study participants. We also gave participants the opportunity to ask questions about the study. Initial eligibility and group classification were screened at this stage, and definitive assignment to the TGD or detransition group was confirmed during the assessment sessions based on participants’ narratives.

Next, they were asked to sign an online consent form, which included a question asking for permission to audio record the semi-structured interview, after which meetings were scheduled. All data were stored and handled in accordance with the protocol approved by the university’s Ethics Committee and the Register of Personal Data Processing. Participants were given the option to choose between a face-to-face or online format for their participation. For participants who chose online participation, we used Psypocket, a platform developed by the General Council of Psychology in Spain which complies with the Organic Law on the Protection of Personal Data. To use Psypocket, participants were given a unique, randomly generated username and password to connect to the platform. Prior to the first interview session, participants were sent two short online surveys asking questions about their sociodemographic information and gender trajectories. Due to the extensive nature of the larger project’s data collection protocol, the assessment was conducted over two separate face-to-face sessions. The first session was dedicated to qualitative component of the study, the semi-structured interview, which covered gender-related development, experiences, and mental health, and ranged from one to four hours. The second session involved completing the quantitative component of the study, the self-report questionnaires. Both sessions averaged two hours each and were conducted by a single investigator (the first author), who is a licensed general health psychologist with experience in transgender health.

### Measures

All the sociodemographic, gender trajectory, and mental health data presented in this study were collected through participant self-report measures during the assessment process.

#### Sociodemographic Variables

Participants were asked questions about their age, sex assigned at birth, current gender self-conceptualization, sexual orientation, country of residence, civil status, educational level, domestic environment, employment status, socio-economic status, and life beliefs. For sexual orientation, participants were simply asked to define it, allowing them to respond from their current gender self-conceptualization. They were given predetermined options to choose from within each question, developed by the research team through an iterative process. However, for current gender self-conceptualization, sexual orientation, employment status, and life beliefs, participants had an additional write-in response option that allowed them to provide an alternative answer.

#### Gender Trajectory Variables

We asked participants about the age at which they had begun their gender transition, the types of steps they had taken to affirm their gender (social, administrative, hormonal, and surgical), and whether they had ever experienced any side or unwanted effects associated with GAHT and/or GAS. Participants were given pre-defined options to choose from within each question, as well as an additional write-in response option that allowed them to personalize their answer. Based on the information provided by participants during the assessment, we were able to obtain additional information about self-prescribed GAHT, other gender-affirming treatments that they had received (e.g., laser hair removal), the approximate duration of GAHT, and whether they had any interest in further medical transition- or detransition-related interventions. For participants who formed the detransition group, we additionally sought to identify the steps they had taken to detransition (social, administrative, hormonal, and surgical), as well as the time that elapsed between the start of their gender transition and the moment where they experienced a change in their gender self-conceptualization.

#### Mental Health Variables

During the interviews, we asked participants about their mental health experiences. Specifically, we asked whether (1) they had ever been in contact with a mental health professional, at what age this contact first occurred, and if this first contact was related to their gender transition process; (2) they had ever received a psychiatric diagnosis and, if so, at what age they received the first diagnosis; and (3) they had taken any type of psychopharmacological medication, past or present. It should be noted that we did not have access to participants’ clinical records, so we decided to distinguish between formally diagnosed psychiatric diagnoses, based on participants’ self-disclosure of a formal diagnosis communicated by a mental health professional or appearing in a psychological/psychiatric report, and other general mental health concerns or difficulties self-reported by participants, either non-clinical or not associated with a formal diagnosis.

During the assessment, some participants self-disclosed experiences of non-suicidal self-injury, suicide attempts, psychiatric admissions, as well as psychosocial stressors and other life adverse experiences including bullying, childhood maltreatment, and sexual abuse. We therefore decided to search for and extract this information from all participants by conducting a detailed review of their interviews and the handwritten notes taken by the interviewer during the assessment. We also tried to determine whether these events had happened before or after the start of gender transition.

### Data Analysis

Data were extracted from the pre-assessment short surveys, the semi-structured interviews, and the handwritten notes taken by the interviewer during the whole assessment. To improve this process, we performed an extensive keyword search using MAXQDA 24 (VERBI Software, 2024). Participants’ write-in responses to the surveys were recoded into pre-existing categories where possible. Write-in responses with four or more counts were grouped into a new category, while responses with fewer than four counts were grouped into an “Other” category. For the mental health variables and some of the gender trajectory variables that we extracted from the interviews and handwritten notes, we created new categories that could be processed statistically. In the absence of access to participants’ formal clinical records, some of these variables were difficult to determine with certainty due to lack of clarity, ambiguity, or inconsistency in the participants’ narratives. Therefore, some of the values are approximations or estimates based on a careful analysis of their stories, as indicated by the symbol ± .

Once all the data were cleaned and coded, we carried out descriptive and inferential statistical analyses using a combination of RStudio (version 4.4.1; Posit Team, 2024) and IBM SPSS Statistics (version 30; IBM Corp., 2024). Specifically, we used means, standard deviations, and ranges for quantitative variables, and frequencies and percentages for qualitative variables. Differences between the TGD and detransition groups were analyzed using tests of association for categorical variables and two-sample *t*-tests for continuous variables. We set a conventional confidence level of 95% (i.e., *α* = .05) to test for statistical significance. We report effect sizes and their 95% confidence intervals (CI) as recommended by Cumming (2012). Percentages were rounded to the first decimal place. Test statistics, effect sizes, and CIs were rounded to the second decimal place, and *p*-values were rounded to the third decimal place.

For the tests of association, we followed the work of Fagerland et al. (2017). Thus, we performed a Suissa-Shuster exact unconditional test for 2 × 2 contingency tables with the ‘Exact’ R package (version 3.3; Calhoun, 2024) using the *z-pooled* (*z*_*p*_) statistic. We report the *difference between proportions* (indicated by *Δ*) as the effect size along with the Agresti-Min exact unconditional 95% CI. For contingency tables larger than 2 × 2, we performed a Pearson chi-squared exact test with the ‘Exact Tests’ module in SPSS. We report Cramer’s *V* as the effect size along with bias-corrected and accelerated (BCa) bootstrap 95% CI calculated using the ‘confintr’ R package (version 1.0.2; Mayer, 2023). For the two-sample *t*-tests, we followed the recommendations of various authors (Delacre et al., 2017, 2021; Karch, 2021; Noguchi et al., 2021) and conducted a permutated Welch’s *t*-test using the ‘MKinfer’ R package (version 1.2; Kohl, 2024). We computed Hedge’s *g* as the effect size along with BCa bootstrap 95% CI using the ‘Durga’ R package (version 2.0; Khan & McLean, 2024). The code we used to perform the statistical analyses is publicly available on the Open Science Framework at https://osf.io/b457r/overview?view_only=47d15ec3dc7543a5b2f0a0a67303a419.

## Results

Below we present data for the full sample of 51 participants. Differences between the TGD and detransition groups are included in the tables. References to the results of each subgroup are noted where appropriate. Of the total 51 participants, 29 formed the TGD group and 22 formed the detransition group (see Fig. 1). For an overview of the gender trajectories of participants within each group, see Tables 1 and 2.

**Table 1:** Gender trajectories of the TGD group (*n* = 29)

ID	Age	Sex assigned at birth	Current GSC	Type of gender transition				GAHT discontinuation
				Social	Hormonal	Administrative	Surgical	
1	47	F	M	X	–	–	–	NA
2	25	F	NB	X	–	–	–	NA
3	19	F	NB	X	–	–	–	NA
4	20	F	NB	X	–	–	–	NA
5	25	F	NB	X	–	–	–	NA
6	30	F	NB	X	X	–	–	–
7	21	F	M	X	X	–	–	–
8	19	F	M	X	X	X	–	–
9	24	F	M	X	X	X	–	–
10	23	F	M	X	X	X	–	–
11	38	F	M	X	X	X	–	–
12	31	F	M	X	X	X	–	–
13	41	M	F	X	X	X	–	–
14	27	F	M	X	X	X	–	–
15	20	M	F	X	X	X	–	–
16	21	F	M	X	X	X	–	–
17	18	M	F	X	X	X	–	–
18	25	F	M	X	X	X	X	–
19	28	F	M	X	X	X	X	–
20	39	M	F	X	X	X	X	–
21	27	M	F	X	X	X	X	–
22	27	F	M	X	X	X	X	–
23	35	M	F	X	X	X	X	–
24	43	M	F	X	X	X	X	–
25	35	M	F	X	X	X	X	–
26	44	F	M	X	X	X	X	–
27	23	F	M	X	X	X	X	–
28	23	F	M	X	X	X	X	X
29	22	F	M	X	X	X	X	X

* F* Female,* M* Male,* NB* Nonbinary,* GSC* Gender self-conceptualization,* GAHT* Gender-affirming hormonal treatment,* NA* Not applicable. An X indicates that the characteristic is present in the participant, while a —indicates that it is not present

**Table 2:** Gender trajectories of the detransition group (*n* = 22)

ID	Age	SAAB	GSC after transition	Current GSC	Type of gender transition				GAHT discontinuation	Reversal of transition-related steps			
					Social	Hormonal	Admin	Surgical		Social	Adm	Hormonal	Surgical
30	20	F	M	F	X	–	–	–	NA	X	NA	NA	NA
31	46	M	F	M	X	–	–	–	NA	X	NA	NA	NA
32	18	F	M	F	X	–	X	–	NA	X	–	NA	NA
33	21	F	M	F	X	X	–	–	X	X	NA	NA	NA
34	19	F	M	F	X	X	–	–	X	X	NA	NA	NA
35	34	M	F	M	X	X	–	–	X	X	NA	NA	NA
36	29	F	M	F	X	X	–	–	X	X	NA	NA	NA
37	27	M	F	NB	X	X	X	–	X	–	–	NA	NA
38	26	F	M	NB	X	X	X	–	X	–	–	NA	NA
39	20	F	M	Q	X	X	X	–	X	–	–	NA	NA
40	19	M	F	Q	X	X	X	–	X	–	–	NA	NA
41	19	F	M	Q	X	X	X	–	X	–	–	NA	NA
42	25	F	M	NB	X	X	X	X	X	–	–	NA	–
43	27	F	M	NB	X	X	X	X	X	–	–	NA	–
44	37	M	F	M	X	X	X	X	X	X	–	–	–
45	34	M	F	M	X	X	X	X	X	X	–	X	–
46	29	F	M	F	X	X	X	X	X	X	X	NA	–
47	23	F	M	F	X	X	X	X	X	X	X	NA	–
48	41	M	F	M	X	X	X	X	X	X	X	NA	–
49	31	M	F	M	X	X	X	X	X	X	–	NA	X
50	49	F	M	F	X	X	X	X	X	X	X	X	–
51	38	F	M	F	X	X	X	X	X	X	X	X	X

* SAAB* Sex assigned at birth,* F* Female,* M* Male,* NB* Nonbinary,* Q* Questioning,* GSC* Gender self-conceptualization,* GAHT* Gender-affirming hormonal treatment, *Admin/Adm* Administrative, *NA* Not applicable. An X indicates that the characteristic is present in the participant, while a — indicates that it is not present

### Sociodemographic Variables

A detailed breakdown of all sociodemographic variables and group comparisons is presented in Table 3. There were no statistically significant differences between the TGD and detransition groups in any of the variables studied, suggesting similarity in terms of their sociodemographic characteristics.

**Table 3:** Sociodemographic characteristics of the study sample (*N* = 51)

	TGD group (*n* = 29)	Detransition group (*n* = 22)	Test statistic	*p*	Effect size [95% CI]
Age, years					
Mean (*SD*)	28.28 (8.42)	28.73 (9.12)	0.18	.855	− 0.05 [− 0.59, 0.53]
Range	18–47	18–49			
Age range, *n* (%)			1.81	.654	0.19 [0.00, 0.30]
18–24	12 (41.4)	8 (36.4)			
25–34	9 (31)	9 (40.9)			
35–44	7 (24.1)	3 (13.6)			
45 +	1 (3.4)	2 (9.1)			
Sex assigned at birth, *n* (%)			− 0.67	.559	− 0.09 [− 0.35, 0.17]
Male	8 (27.6)	8 (36.4)			
Female	21 (72.4)	14 (63.6)			
Current GSC, *n* (%)			6.88	.073	0.37 [0.00, 0.52]
Man	16 (55.2)	6 (27.3)			
Woman	8 (27.6)	9 (40.9)			
Nonbinary	5 (17.2)	4 (18.2)			
Questioning	0 (0)	3 (13.6)			
Sexual orientation,^a^* n* (%)			3.67	.314	0.27 [0.00, 0.38]
Heterosexual	11 (37.9)	5 (22.7)			
Homosexual	2 (6.9)	2 (9.1)			
Bi + (bi/pansexual, fluid)	16 (55.2)	13 (59.1)			
Questioning	0 (0)	2 (9.1)			
Country of residence, *n* (%)			1.34	.209	0.10 [− 0.06, 0.31]
Spain	28 (96.6)	19 (86.4)			
Latin America^b^	1 (3.4)	3 (13.6)			
Living environment, *n* (%)			− 1.65	.112	− 0.18 [− 0.40, 0.05]
Urban	21 (72.4)	20 (90.9)			
Rural	8 (27.6)	2 (9.1)			
Civil status, *n* (%)			1.57	.487	0.18 [0.00, 0.33]
Single	15 (51.7)	8 (36.4)			
Married/with partner	12 (41.4)	13 (59.1)			
Separated/divorced	2 (6.9)	1 (4.5)			
Education, *n* (%)			1.41	.723	0.17 [0.00, 0.26]
Primary school	1 (3.4)	2 (9.1)			
Secondary/high school	8 (27.6)	8 (36.4)			
Vocational training	8 (27.6)	5 (22.7)			
University	12 (41.4)	7 (31.8)			
Employment status, *n* (%)			1.35	.865	0.16 [0.00, 0.28]
Student	7 (24.1)	8 (36.4)			
Unemployed	7 (24.1)	5 (22.7)			
Self-employed	4 (13.8)	3 (13.6)			
Part-time job	4 (13.8)	3 (13.6)			
Full-time job	7 (24.1)	3 (13.6)			
Domestic situation, *n* (%)			3.37	.539	0.26 [0.00, 0.35]
Living with parents	13 (44.8)	11 (50)			
Living with a partner	6 (20.7)	6 (27.3)			
Living with roommates	2 (6.9)	3 (13.6)			
Living alone	2 (6.9)	1 (4.5)			
Living with other relatives	6 (20.7)	1 (4.5)			
Socioeconomic status, *n* (%)			2.35	.720	0.21 [0.00, 0.29]
No income	7 (24.1)	9 (40.9)			
< 500€	9 (31)	4 (18.2)			
500–1,000€	6 (20.7)	3 (13.6)			
1,000–2,000€	6 (20.7)	5 (22.7)			
> 2,000€	1 (3.4)	1 (4.5)			
Life beliefs, *n* (%)			2.89	.428	0.24 [0.00, 0.38]
Non-believer	19 (65.5)	13 (59.1)			
Agnostic	3 (10.3)	1 (4.5)			
Catholic	4 (13.8)	2 (9.1)			
Other (e.g., Buddhism)	3 (10.3)	6 (27.3)			

* GSC* Gender self-conceptualization

^a^Sexual orientation categories are based on participants’ current gender self-conceptualization. The corresponding results by sex assigned at birth for the TGD group are: 2 heterosexual (1 male, 1 female), 11 homosexual (6 male, 5 female), and 16 bi + (bi/pansexual, fluid) (1 male, 15 female). The corresponding results by sex assigned at birth for the detransition group are: 5 heterosexual (1 male, 4 female), 13 bi + (bi/pansexual, fluid) (6 male, 7 female), 2 homosexual (1 male, 1 female), and 2 questioning (2 female)

^b^Specific countries are not disclosed to protect participant anonymity due to the small number of participants

The average age of the participants was 28.47 years (*SD* = 8.64, range 18–49). Three quarters of the sample were aged 18–24 years (39.2%) and 25–34 years (35.3%). More than two thirds of the sample (68.6%) were AFAB, while 31.4% were AMAB. In terms of current gender self-conceptualization, 43.1% of the sample defined themselves as men, 33.3% as women, and 23.5% as nonbinary or questioning. Over half of the participants (56.9%) were bisexual, pansexual, or fluid. Although 92.2% of the participants lived in Spain, seven were originally born in other countries, mainly in Latin America. Almost half of the participants (49%) had a partner or were married, and the majority (62.7%) reported their highest level of education as below university level. At the time of the assessment, 29.4% of participants were students and 23.5% were unemployed. In terms of socioeconomic status, 31.4% reported no personal income and 43.1% reported an income of €1,000 or less per month.

### Gender Trajectory Variables

A detailed breakdown of gender trajectory variables and group comparisons is provided in Table 4.

**Table 4:** Gender trajectory variables in the study sample (*N* = 51)

	TGD group (*n* = 29)	Detransition group (*n* = 22)	Test statistic	*p*	Effect size [95% CI]
Social transition, *n* (%)	29 (100)	22 (100)	0.00	1.000	0.00 [− 0.11, 0.16]
Age at social transition, years					
Mean (*SD*)	± 22.62 (± 8.21)	± 18.86 (± 6.88)	− 1.78	.078	0.49 [− 0.10, 1.02]
Range	± 13–45	± 11–39			
Age range, *n* (%)			4.97	.432	0.31 [0.00, 0.38]
0–11	0 (0)	1 (4.5)			
12–17	9 (31)	11 (50)			
18–24	10 (34.5)	7 (31.8)			
25–34	7 (24.1)	2 (9.1)			
35–44	2 (6.9)	1 (4.5)			
45 +	1 (3.4)	0 (0)			
Administrative transition, *n* (%)	22 (75.9)	16 (72.7)	0.25	.840	0.03 [− 0.22, 0.30]
Medical transition, *n* (%)	24 (82.8)	19 (86.4)	− 0.35	.790	− 0.04 [− 0.24, 0.19]
Age at medical transition, years					
Mean (*SD*)	± 23.58 (± 6.26)	± 19.68 (± 4.37)	− 2.40	**.017**	0.71 [0.08, 1.25]
Range	± 16–40	± 14–39			
GAHT, *n* (%)	24 (100)	19 (100)	0.00	1.000	0.00 [− 0.14, 0.18]
Duration, years					
Mean (*SD*)	± 4.85 (± 4.56)	± 5.95 (± 6.48)	0.63	.527	− 0.19 [− 0.77, 0.46]
Range	± 0.5–16	± 0.17–20			
Self-prescribed, *n* (%)	1 (4.2)	4 (21.1)	− 1.72	.102	− 0.17 [− 0.41, 0.04]
Side/unwanted effects, *n* (%)	13 (54.2)	16 (84.2)	− 2.09	**.043**	− 0.30 [− 0.54, − 0.01]
Emotional instability/difficulties	10 (76.9)	11 (68.8)			
Physical pain/discomfort	8 (61.5)	8 (50)			
Weakness/fatigue	4 (30.8)	4 (25)			
Excessive/low sex drive	4 (30.8)	3 (25)			
Skin, hair, sweat, and body odor changes	2 (13.3)	2 (12.5)			
Androgenic alopecia	3 (20)	1 (6.3)			
Other (e.g., hot flushes)	7 (46.7)	9 (56.3)			
Discontinuation	2 (8.3)	19 (100)	− 5.97	** < .001**	− 0.92 [− 0.99, − 0.72]
GAS, *n* (%)	12 (50)	10 (52.6)	− 0.17	.892	− 0.03 [− 0.32, 0.27]
Adverse outcomes/complications after GAS, *n* (%)	5 (41.7)	7 (70)	− 1.33	.208	− 0.28 [− 0.62, 0.15]
Dissatisfaction with cosmetic results	3 (60)	3 (42.9)			
Loss of sensation	0 (0)	4 (57.1)			
Other (e.g., stenosis)	4 (80)	4 (57.1)			
Other procedures (e.g., hair removal), *n* (%)	7 (29.2)	7 (36.8)	− 0.53	.620	− 0.08 [− 0.36, 0.21]
Interest in further interventions, *n* (%)	21 (72.4)	5 (22.7)	3.52	** < .001**	0.50 [0.22, 0.70]

* GAHT* Gender-affirming hormonal treatment,* GAS* Gender-affirming surgery,* NA* Not applicable. The ± symbol indicates that the value is an approximation or estimate based on the analysis of participant narratives. Bold indicates a statistically significant difference (*p* < .05)

#### Social and Administrative Transition

All 51 participants reported having initiated a social transition. The average age at which participants started socially transitioning was ± 21.00 years (*SD* =  ± 7.82, range ± 11–45), 39.2% during adolescence and 58.8% during adulthood, mostly between the ages of 18–24 years (33.3%) and 25–34 years (17.6%). The age at social transition was lower for the detransition group (*M* = 18.86) than for the TGD group (*M* = 22.62), though this difference was not statistically significant (*p* = .078). Some participants began their social transition in online environments, while others reported experiences of being treated by others as their target gender prior to their social transition. As part of their social transition (and prior to GAHT or GAS for those who underwent it), many AFAB participants in both groups reported chest binding and some reported packing, while some AMAB participants reported tucking and/or bra stuffing. Most participants (74.5%) also transitioned administratively. Of the seven participants in the TGD group who had not transitioned administratively, three (42.9%) were in the process. No significant difference was found between groups in the rate of administrative transition.

#### Medical Transition: Gender-Affirming Hormone Therapy and Gender-Affirming Surgery

A majority of the sample (84.3%) reported medical transition, with no significant difference in rates between the TGD group (82.8%) and the detransition group (86.4%). The mean age at which participants began medical transition was ± 21.86 years (*SD* =  ± 5.78, range ± 14–40). A statistically significant difference was found between groups, with detransitioning participants starting medical transition at a younger age (*M* =  ± 19.68) compared to TGD participants (*M* =  ± 23.58) (*p* = .017, Hedge’s *g* = 0.71 [95% CI 0.08, 1.25]). At least four participants (7.8%; two in each group) began medical transition before social transition.

Of the 43 participants who reported medical interventions, all had undergone GAHT, for an average of slightly over five years (*M* =  ± 5.34 years, *SD* =  ± 5.45, range ± 0.17–20). Despite the difference in the age at the start of medical transition, no statistically significant difference was found between the groups regarding the duration of GAHT (*p* = .527). Only two participants (one in each group) had their puberty suppressed with gonadotropin-releasing hormone analogs before GAHT. Five participants (11.6%) reported self-prescribing GAHT and, of these, one (in the detransition group) never sought professional help. Twenty-nine (67.4%) participants self-reported side or unwanted effects associated with GAHT; a significantly higher proportion of the detransition group (84.2%) reported these effects compared to the TGD group (54.2%) (*p* = .043). The most commonly reported effects were emotional instability/difficulties (72.4%), physical pain/discomfort (55.2%), weakness/fatigue (27.6%), and excessive/low sex drive (24.1%). However, some participants (at least nine in the TGD group and one in the detransition group) who did not report any side effects or adverse effects of GAHT in the short surveys described experiences with GAHT that could be categorized as such during the assessment, including some which other participants reported as side or unwanted effects. At least four participants (two in both groups) reported a history of temporary discontinuation or irregular use of GAHT.

At the time of the assessment, 21 participants (48.8%) reported having discontinued GAHT, two in the TGD group (both citing side effects) and the remaining 19 in the detransition group. This difference in discontinuation rates was statistically significant (*p* < .001). Of these 21, 85.7% discontinued GAHT on their own (all in the detransition group) and the remaining (two in the TGD group and one in the detransition group) did so with the help of their care provider. A significantly higher proportion of the detransition group discontinued GAHT on their own (94.7%) compared to the TGD group (0%) (*z*_*p*_ = − 3.64, *p* = .005, *Δ* = − 0.95 [95% CI − 0.97, − 0.21]), although the majority (77.8%) of those who discontinued on their own eventually informed their providers of their decision to discontinue GAHT.

More than half (51.2%) of the participants who reported medical interventions indicated having undergone GAS, with no significant difference between groups. Top surgery was the most common procedure (81.8%), followed by gonadectomy (31.8%) and bottom surgery (22.7%). Of those with GAS, 54.5% self-reported adverse outcomes or complications, most notably dissatisfaction with cosmetic results (50%) and loss of sensation (33.3%). Similarly to GAHT, at least three participants (two in the TGD group and one in the detransition group) did not report any adverse outcomes or complications of GAS in the short surveys, but during the assessment described experiences that could be categorized as such, including some which other participants reported as adverse outcomes or complications. Three participants in the TGD group were on the waiting list for revision surgery. An additional 32.6% of participants reported having initiated or undergone other procedures, mostly laser hair removal. Two of them reported having undergone facial surgery.

#### Detransition Trajectories and Future Interventions

Focusing specifically on the participants who formed the detransition group (*n* = 22), the mean age at which they experienced a change in their gender self-conceptualization after gender transition was ± 25.46 years (*SD* =  ± 8.50, range ± 15–47), and the mean duration between the start of their gender transition and the time they experienced a change in their gender self-conceptualization was over six years (*M* =  ± 6.75 years, *SD* =  ± 6.00, range ± 1–24.5). The majority of participants in this group (68.2%) reidentified with their sex assigned at birth. Of the remaining seven participants, four (18.2%) shifted from a binary to a nonbinary identity, while three (13.6%) were questioning their TGD identity but without a clear reidentification with their sex assigned at birth. Of the 16 participants in this group who transitioned administratively, 31.3% reported applying for a change or reversal. In addition, of the 19 participants who medically transitioned, seven (36.8%) reported reversal medical procedures: five laser hair removal, three reversal hormonal therapy, and two reversal surgeries (one breast implant removal and one facial surgery). Two participants were on the waiting list for reversal surgery, of which one had already begun reversal hormonal therapy after gonadectomy, and one had initiated the procedure for reversal surgery.

Regarding future plans, 51% of the total sample expressed interest in further medical interventions related to gender transition or detransition. A statistically significant difference was found between groups (*p* < .001), with a higher proportion of the TGD group (72.4%) expressing interest in further interventions compared to the detransition group (22.7%). This most notably included top surgery in the TGD group and reversal surgery in the detransition group. At least six participants in the TGD group were already on the waiting list for GAS or had initiated the procedure to undergo the surgery. 

### Mental Health Variables

A detailed breakdown of all mental health variables for both groups is presented in Table 5. There were no statistically significant differences between the TGD and detransition groups in any of the variables studied, suggesting similarity in terms of their mental health-related history.

**Table 5:** Mental health variables in the study sample (*N* = 51)

	TGD group (*n* = 29)	Detransition group (*n* = 22)	Test statistic	*p*	Effect size [95% CI]
Contact with a mental health professional, *n* (%)	29 (100)	21 (95.5)	1.16	.339	0.05 [− 0.08, 0.23]
Mean age at first contact (*SD*), years	± 20.59 (± 7.86)	± 16.67 (± 6.61)	− 1.91	.061	0.53 [− 0.05, 1.08]
Range	± 8–41	± 8–36			
Formal psychiatric diagnosis, *n* (%)	17 (58.6)	10 (45.5)	0.93	.377	0.13 [− 0.15, 0.39]
Mean age at first diagnosis (*SD*), years	± 17.41 (± 4.49)	± 20.20 (± 8.11)	1.00	.341	− 0.40 [− 1.16, 0.49]
Range	± 11–28	± 10–34			
Number of formal diagnoses, *n*					
Mean (*SD*)	± 2.12 (± 0.99)	± 2.30 (± 1.25)	0.39	.670	− 0.16 [− 0.92, 0.68]
Range	± 1–4	± 1–5			
Categories of formal diagnoses, *n* (%)			–	–	–
ASD/ADHD	4 (23.5)	4 (40)			
Emotional disorder	13 (76.5)	8 (80)			
Obsessive–compulsive-related disorder	1 (5.9)	1 (10)			
Post-traumatic stress disorder	1 (5.9)	1 (10)			
Eating/substance abuse disorder	4 (23.5)	0 (0)			
Bipolar/psychotic/personality disorder	3 (17.6)	3 (30)			
Non-suicidal self-injury, *n* (%)	7 (24.1)	4 (18.2)	0.51	.636	0.06 [− 0.19, 0.29]
Suicidal ideation, *n* (%)	16 (55.2)	12 (54.5)	0.04	.977	0.01 [− 0.27, 0.28]
Suicide attempt, *n* (%)	7 (24.1)	4 (18.2)	0.51	.636	0.06 [− 0.19, 0.29]
Psychiatric admission, *n* (%)	5 (17.2)	1 (4.5)	1.39	.186	0.13 [− 0.08, 0.31]
Use of psychopharmacological medication					
Lifetime, *n* (%)	16 (55.2)	13 (59.1)	− 0.28	.809	− 0.04 [− 0.30, 0.24]
Antidepressants	10 (62.5)	12 (92.3)			
Anxiolytics/hypnotics	12 (75)	8 (61.5)			
Antipsychotics/antiepileptics/mood stabilizers	7 (43.8)	2 (15.4)			
Other (amphetamines and cannabinoids)	1 (6.3)	1 (7.7)			
Current, *n* (%)	5 (17.2)	8 (36.4)	− 1.55	.152	− 0.19 [− 0.44, 0.06]
Antidepressants	3 (60)	6 (75)			
Anxiolytics/hypnotics	3 (60)	3 (37.5)			
Antipsychotics/antiepileptics/mood stabilizers	1 (20)	2 (35)			
Other (amphetamines and cannabinoids)	1 (20)	1 (12.5)			
Experiences of bullying, *n* (%)	13 (44.8)	12 (54.5)	− 0.69	.520	− 0.10 [− 0.37, 0.19]
Experiences of childhood maltreatment, *n* (%)	3 (10.3)	2 (9.1)	0.15	.963	0.01 [− 0.20, 0.20]
Experiences of sexual abuse, *n* (%)	6 (20.7)	4 (18.2)	0.22	.846	0.03 [− 0.21, 0.25]

The ± symbol indicates that the value is an approximation or estimate based on the analysis of participant narratives

All but one participant (98%) reported having had contact with a mental health professional during their lifetime, for the first time at an average age of ± 18.94 years (*SD* =  ± 7.55, range ± 8–41). The mean age at first contact was lower for the detransition group. For those participants who had been in contact with a mental health professional, this first contact was reported to be related to the gender transition process in 34% of cases. Slightly over half of the sample (52.9%) reported formal psychiatric diagnoses other than GD, first received at an average age of ± 18.44 years (*SD* =  ± 6.09, range ± 10–34). Participants in the detransition group received the first diagnosis at a later age compared to the TGD group. The average number of lifetime formal diagnoses received was ± 2.19 (*SD* =  ± 1.08, range ± 1–5). Emotional disorders (e.g., depression, anxiety) were most reported (77.8%), followed by ASD/ADHD (29.6%) and bipolar, psychotic, and personality disorders (22.2%). In addition, one participant (in the TGD group) reported having been formally assessed as intellectually gifted.

Most participants (94.1%) also self-disclosed other general mental health concerns or difficulties during their lifetime (non-clinical or not associated with a formal diagnosis), most frequently related to depression and/or anxiety (70.8%), disordered eating (47.9%), sleep problems (27.1%), and obsessive–compulsive-related problems (22.9%; e.g., body dysmorphia). Six participants (four in the TGD group and two in the detransition group) suspected having ASD and one participant in the TGD group suspected having ADHD. Furthermore, 21.6% self-disclosed non-suicidal self-injurious behaviors, in all cases before gender transition. Suicidal ideation was self-disclosed by 54.9% of participants and 21.6% self-disclosed at least one suicide attempt (13.7% more than one, five in the TGD group and two in the detransition group), in 54.5% of cases before gender transition. An additional 11.8% of participants self-disclosed a history of psychiatric admission, in half of the cases (50%) before gender transition. Over half of the participants (56.9%) reported a history of psychopharmacological medication use during their lifetime, although only 25.5% were currently receiving such treatment. Antidepressants and anxiolytics/hypnotics were the most reported types of psychopharmacological medication (75.9% and 69%, respectively).

In terms of psychosocial stressors and adverse life experiences, almost half of the sample (49%) self-disclosed a history of experiences of bullying. In 92% of cases, this occurred during childhood and/or adolescence before gender transition (the remaining occurred during gender transition) and, in 48% of cases, participants explicitly mentioned instances of homophobic bullying or bullying related to visible gender nonconformity (seven participants in the TGD group and five in the detransition group). Although many participants mentioned family conflicts and/or instances of maltreatment from family members at various points in their lives, five (9.8%) explicitly self-disclosed a history of repeated psychological and physical abuse. In all cases, this occurred prior to gender transition, specifically during childhood and adolescence, from one or both parents. In addition, two participants in the TGD group self-disclosed parental abandonment during childhood, and another two experiencing intimate partner violence (one before and one after gender transition). One participant in the TGD group was kicked out of the family home after initiating the gender transition process, and two participants (both in the TGD group) explicitly mentioned having suffered a direct physical attack during their gender transition. Experiences of sexual abuse were self-disclosed by 19.6% of participants (60% prior to gender transition), with four cases (two participants in each group) occurring during childhood or adolescence. One participant in the TGD group self-disclosed an experience of sexual orientation conversion therapy during adolescence.

## Discussion

In this article, we examined and compared the sociodemographic, gender trajectory, and mental health characteristics of a convenience sample of adult Spanish-speaking individuals with different gender transition pathways. Overall, the findings illustrate the heterogeneity of trajectories and outcomes following gender transition, contributing to our understanding of the diversity inherent in these experiences. The lack of statistically significant differences between the groups in most of the variables studied suggests that there are no clear indicators or patterns to distinguish between those who will detransition and those who will not, making the prediction of future gender trajectories a very difficult (if not impossible) task (Coleman et al., 2022; Expósito-Campos et al., 2023c). Instead, care services must be developed around a flexible, person-centered approach that accommodates this diversity and supports individuals through evolving gender self-conceptualizations, acknowledging that widely different outcomes are possible for individuals with similar life histories.

### Sociodemographic Variables

With respect to their sociodemographic characteristics, similar to previous research conducted with both non-probabilistic (e.g., Fisher et al., 2024; James et al., 2016, 2024) and representative samples (e.g., Krueger et al., 2020; Twenge et al., 2025) of TGD people, as well as survey- and interview-based studies with individuals who have detransitioned (Haarer, 2022; Littman, 2021; Littman et al., 2024; MacKinnon et al., 2022; Maragos et al., 2025; O’Donnell, 2023; Pullen Sansfaçon et al., 2023; Vandenbussche, 2022), we observed that a majority of participants were AFAB. In addition, most had started their gender transition in adolescence or young adulthood. These findings likely reflect the broader sociodemographic trends that have been reported in international epidemiological and clinic-based research on people who self-identity as TGD or seek gender-affirming care (Sun et al., 2023; Twenge et al., 2025; Zhang et al., 2020, 2021). We also found that many participants defined themselves as bisexual, pansexual, or fluid with regards to their sexual orientation, similar to previous research with individuals who are TGD or have experiences of detransition (Belza et al., 2024; James et al., 2016; Littman, 2021; MacKinnon et al., 2022; McKenna et al., 2024; Reisner et al., 2023).

A majority of our study participants had a low level of education and socioeconomic status. These findings are largely consistent with those from other international reports (e.g., Barrientos Delgado et al., 2021; Fisher et al., 2024; James et al., 2016; Krueger et al., 2020), as well as the preliminary results from the largest Spanish study of TGD people to date, Transaludes, with 1,823 respondents (Belza et al., 2024). In the Transaludes study, 41.6% of participants were young adults, just over half reported not having university studies, and more than half defined their socioeconomic situation as “tight” or “bad/very bad.”

### Gender Trajectory Variables

In terms of their gender trajectory variables, all of our study participants had transitioned socially, with many reporting non-medical gender affirming practices such as chest binding or tucking (see Diana et al., 2026). We also observed high rates of administrative and medical transition. These rates are consistent with the results of the Transaludes study (Belza et al., 2024), in which about three quarters of binary TGD participants had changed their name or gender marker, 80.1% had medically transitioned with GAHT, and 35.7% had undergone GAS. However, our figures are higher than in other international studies (Barrientos Delgado et al., 2021; Fisher et al., 2024; James et al., 2016; Lane et al., 2022), likely reflecting sampling differences.

Looking at the age of participants at the start of gender transition, we observed that those in the detransition group started socially transitioning at a younger age, with a difference of about four years compared to the TGD group. They also started medical transition about four years earlier than the TGD group, a difference that was statistically significant. While this may suggest that individuals who transition at a younger age are more likely to detransition or experience changes in their gender self-conceptualization, findings from other studies are inconclusive in this regard. For example, Butler et al. (2022) found higher rates of detransition among adolescents younger than 16 years of age compared to those 16 years and older at endocrine referral, and Gómes-Porras et al. (2020) found that individuals 18 years of age or older at the start of gender transition were less likely to detransition. However, Roberts et al. (2022) found higher rates of GAHT discontinuation in individuals who began medical transition as adults compared to those who began as minors.

We also observed that the average time between the start of gender transition and the change in gender self-conceptualization in our study was over six years, with some participants detransitioning after over 20 years, which is higher than the figures reported in other previous studies (e.g., 3.9 years in Littman, 2021; 5.4 years in Littman et al., 2024; 4.7 years in Vandenbussche, 2022). Interestingly, the mean age at which our participants experienced a change in their gender self-conceptualization was ± 25.46, which is broadly consistent with findings from developmental psychology that identity development is an ongoing process that can occur in the late twenties (e.g., Carlsson et al., 2015). While this discrepancy may be related to sampling differences, the findings highlight the importance of long-term follow-up to understand gender transition pathways and outcomes among TGD individuals.

Regarding the nine nonbinary participants in the study, only one of the five in the TGD group had medically transitioned with GAHT, and all four in the detransition group had discontinued GAHT. Although the number of nonbinary TGD participants in our study was small, the results overall reflect a trend toward less medicalized gender transitions among nonbinary individuals compared to binary TGD individuals (e.g., Belza et al., 2024; Cheung et al., 2020; Chew et al., 2020; Reisner & Hughto, 2019), as well as the intersection between being nonbinary and detransitioning (Nieder, 2023). In this regard, nonbinary and detransitioning experiences may overlap, as both involve evolving understandings of gender and ongoing identity development (Nieder, 2023). In addition, none of the nonbinary participants in the TGD group reported changing their administrative documents, which is consistent with the low rate of administrative gender transition among nonbinary participants in the Transaludes study (Belza et al., 2024). The fact that some participants began medical transition before social transition highlights the non-linear nature of some gender transition processes (see Rachlin, 2018).

Regarding medical transition, there were statistically significant differences between the groups with regards to self-reported side or unwanted effects associated with GAHT, with 30% more participants in the detransition group reporting such effects. Although the differences for GAS were not statistically significant, there was a 28% difference in the proportion of participants who reported adverse outcomes or complications in the detransition group compared to the TGD group. While most of the reported side effects are well-known and expected from GAHT (Prince & Safer, 2020), and the reported adverse outcomes/complications of GAS have been previously discussed in the literature (Oles et al., 2022a, 2022b), this finding is consistent with previous research indicating that unsatisfactory outcomes and health complications following medical gender transition may contribute to the decision to detransition (Expósito-Campos et al., 2023c; Pazos Guerra et al., 2022).

An interesting observation was that, for a significant number of participants (the majority of whom were in the TGD group), there was an incongruence between what they reported in the short surveys and what they reported in the interviews both in terms of side or unwanted effects associated with GAHT and adverse outcomes or complications following GAS. While this may be related to participants having a different understanding of what constitutes a side effect or complication, which in turn affects their reporting of such events, there is also literature showing that patients’ expectations and previous experiences, the informed consent process and level of communication with and trust in the provider, or the severity and familiarity of treatment outcomes can influence their categorization as side effects or complications (Black et al., 2014; Faasse & Petrie, 2013; Waters et al., 2016). However, it may also be the case that more positive transition experiences overall influence participants’ level of satisfaction and thus how side effects or complications are perceived.

In relation to GAHT specifically, it is also worth noting that the number of participants who self-prescribed GAHT was higher in the detransition group compared to the TGD group (although not statistically significant). Of the five participants who reported self-prescribing, four (80%) were AMAB. Although we did not specifically investigate the reasons for self-prescription of GAHT among these participants, previous studies have reported that it is not uncommon, is significantly more prevalent among AMAB people, and is usually associated with barriers to care or a desire for faster medical transition (Kennedy et al., 2021; Mepham et al., 2014; Rotondi et al., 2013). The prevalence rate of 11.6% of self-prescribed GAHT in our study is lower than in other studies (Mepham et al., 2014; Rotondi et al., 2013), but slightly higher than the overall self-prescription rate of 7.4% in the Transaludes study (14.7% among transfeminine participants; Belza et al., 2024). This may be due to sampling differences or different patterns of self-reporting.

The statistically significant difference in the number of participants who discontinued GAHT in each group was also unsurprising and expected, as discontinuation or reversal of gender-affirming medical care is frequent in detransition experiences. Therefore, this finding reinforces the association between a shift in gender self-conceptualization and the subsequent decision to stop GAHT within our sample. However, the fact that 8.3% of participants in the TGD group also discontinued GAHT shows that this may not be an uncommon experience and that it is important to distinguish between identity-motivated and non-identity-motivated GAHT discontinuation. For example, in a survey study conducted in North America (MacKinnon et al., 2024), the authors found that 16.8% of participants reported ever discontinuing GAHT, and only 32% of these reported doing so because of a change in gender identity.

One finding about the detransition group is also worth discussing. We found that a significantly higher proportion of participants in this group discontinued GAHT on their own than in the TGD group, where the two participants who discontinued GAHT did so under the clinical supervision of their providers. Only one participant in the detransition group stopped GAHT under clinical supervision for medical reasons. On the one hand, this observation is consistent with previous studies showing that disengagement or avoidance behaviours are common among people who detransition (Gelly et al., 2025; Haarer, 2022; Littman, 2021; Littman et al., 2024; MacKinnon et al., 2022). On the other hand, most of the detransition participants who discontinued GAHT on their own eventually informed their providers of their decision (77.8%), a percentage significantly higher than reported in previous studies (e.g. 24% in Littman, 2021; 27.1% in Littman et al., 2024). However, this may be related to the fact that a number of the detransitioning participants were referred to our study by their gender care providers, which implies knowledge of the participant’s decision to detransition.

There was a statistically significant difference between the TGD and detransition groups in terms of interest in further interventions, with a higher proportion in the TGD group. This was unsurprising and to some extent expected, as many participants in the TGD group were assessed at an “early” stage of their medical transition process. However, it should also be highlighted that only 22.7% of participants in the detransition group indicated that they were interested in further interventions. This, together with the fact that only 31.3% had applied for administrative reversal and 36.8% had reversed some aspect of their medical transition, shows that the experience of detransition does not necessarily involve a complete reversal of all the steps or changes involved in gender transition (Pullen Sansfaçon et al., 2023) or a desire for more treatments.

### Mental Health Variables

In terms of psychosocial stressors and mental health, just over half of the study sample reported having received a formal psychiatric diagnosis (the average was two diagnoses) and more than 90% self-reported other general mental health concerns or difficulties during their lifetime. In addition, more than half of the participants had a history of psychopharmacological medication use. We also observed a self-disclosed rate of over 50% for lifetime suicidal ideation and over 20% for both lifetime non-suicidal self-injury and suicide attempts. Overall, these findings add to the evidence from other studies showing poor mental health in TGD people (Kalayjian et al., 2024; Kohnepoushi et al., 2023; Pinna et al., 2022) and people with an experience of detransition (Gould et al., 2024; Littman, 2021; Littman et al., 2024; Vandenbussche, 2022). Interestingly, participants in the detransition group reported an earlier first contact with a mental health professional, with a mean difference of three years, but a later mean age at first psychiatric diagnosis compared to the TGD group, with a mean difference also of almost three years. This may be explained by the fact that, among those who had contact with a mental health professional, the subset of those who received a psychiatric diagnosis were younger at first contact in the TGD group and older in the detransition group.

In terms of mental health diagnoses and use of psychopharmacological medication, our data are broadly consistent with the findings of the Transaludes study (Belza et al., 2024). However, in this study, the percentage of participants self-reporting non-suicidal self-injury, suicidal ideation, and suicide attempts was significantly higher. Similarly, the number of participants self-disclosing experiences of violence, including of a sexual nature, was higher in this and other studies (e.g., Devís-Devís et al., 2017; Strauss et al., 2020). These discrepancies may be related to social desirability bias, as previous research suggests that interview survey formats may lead to underestimation of mental health problems compared to online self-report (Rickwood & Coleman-Rose, 2023). However, the results may also be explained by the fact that we did not explicitly ask about these experiences, so participants may not have felt the need to spontaneously disclose such sensitive information about themselves. Finally, the high prevalence of self-reported bullying experiences in our sample, and the fact that in many cases this was associated with participants’ presumed sexual orientation and gender nonconforming expression, is also consistent with the findings of a recent systematic review, which found a positive significant association between gender nonconformity and victimization (Hu et al., 2024). Another study found that homophobic bullying was associated with changes in gender identity over time (DeLay et al., 2018), suggesting that, for some participants, these experiences may have played a role in shaping the development of their gender self-conceptualization.

### Strengths and Limitations

To our knowledge, this is the first study to intentionally recruit and compare both TGD and people with an experience of detransition across a range of gender- and mental health-related variables. Although exploratory in nature, it identified both shared and divergent characteristics that may contribute to inform clinical practice and advance potential avenues for future research. We sought to explicitly conceptualize and operationalize our criterion for dividing participants into the TGD and detransition groups in order to contribute to much needed conceptual clarity in the emerging field of detransition research (MacKinnon et al., 2023). In addition, data were collected through a face-to-face in-depth assessment, which were analyzed and interpreted on a case-by-case basis, taking into account the unique experiences, developmental contexts, and needs of each participant.

However, some limitations should also be considered. First, the cross-sectional nature of the study meant that we were not able to assess participants’ trajectories and decisions regarding their gender transition or detransition over time. Secondly, the data presented in this article come from a convenience sample recruited using several non-probability techniques, which means that the findings may not be generalizable to other populations of TGD and detransitioned participants, mainly because of sampling bias. Furthermore, our sample was culturally specific, comprising only Spanish-speaking individuals, most of whom lived in Spain. The social attitudes, healthcare systems, and legal frameworks specific to this context undoubtedly influenced their experiences of transition and detransition. Therefore, our findings may not be applicable to TGD and detransitioning individuals from different cultural backgrounds. Thirdly, the small size of both the overall sample and the two study groups significantly limited the statistical power of our analyses to detect significant differences between them in the study variables. Fourthly, an alternative explanation to the lack of observed statistically significant differences between the groups may be related to not having measured specific relevant variables that could have potentially distinguished them (e.g., attachment style, family dynamics). While our protocol was extensive and based on previous research, this aspect warrants investigation in future studies. Furthermore, we extracted some gender trajectory and mental health-related variables based on a detailed and careful analysis of participants’ narratives and spontaneous self-disclosures, but we did not include questions specifically targeting these aspects and we did not have access to their formal clinical records. As a result, in addition to potential biases in the data due to participant recall bias, some of the values are approximate and may be subject to underreporting.

### Conclusion

This study examined and compared sociodemographic, gender trajectory, and mental health variables between two groups of participants with different gender trajectories after transition. Detransitioning participants started medically transitioning at a younger age compared to TGD participants. In addition, the number of participants who discontinued GAHT and reported side or unwanted effects related to GATH was significantly higher in the detransition group, whereas the number of participants with interest in further interventions was significantly higher in the TGD group. There were no statistically significant differences in any of the other variables, suggesting an overall absence of disparities in life experiences and mental health between the two groups. While the findings highlight and contribute to our understanding of the diversity and complexity of gender transition outcomes and trajectories, as well as the development of gender self-conceptualization across the lifespan, they also underscore the lack of specific indicators to help us predict or anticipate such variability. In addition, the findings add to previous evidence of significant mental health disparities in TGD and detransitioning populations, highlighting the need for the development of specific clinical resources and services to address these issues. Further comparative and longitudinal research is needed to understand the similarities and differences between TGD people and those who detransition, in order to inform and develop mental health care and support tailored to the unique characteristics of these experiences.

## Data Availability

Due to the sensitive nature of the data and the fact that the participants did not give their consent for it to be shared publicly, the data supporting the findings of this study are not available.

## Code Availability

The code used to perform the statistical analyses is available on the Open Science Framework at https://osf.io/b457r/overview?view_only=da9cff1ce2e6423db98ee4d9f3807230.
